# Pediatric Liver and Kidney Transplant Recipients Demonstrate Greater Serological Response to SARS-CoV-2 Vaccination Than Adults

**DOI:** 10.1097/TXD.0000000000001787

**Published:** 2025-04-29

**Authors:** Tobias Laue, Maria Pilar Ballester, Lily Meoli, Carl Grabitz, Eva Uson, Lorenzo D´Antiga, Valerie McLin, Montserrat Pujadas, Ângela Carvalho-Gomes, Ivan Sahuco, Ariadna Bono, Federico D’Amico, Raffaela Viganò, Elena Diago, Beatriz Tormo Lanseros, Elvira Inglese, Dani Martinez Vazquez, Annelotte Broekhoven, Marjolein Kikkert, Shessy P. Torres Morales, Sebenzile K. Myeni, Mar Riveiro-Barciela, Adriana Palom, Nicola Zeni, Alessandra Brocca, Annarosa Cussigh, Sara Cmet, Maria Desamparados Escudero-García, Matteo Stocco, Leonardo Antonio Natola, Donatella Ieluzzi, Veronica Paon, Angelo Sangiovanni, Elisa Farina, Clara Dibenedetto, Yolanda Sánchez-Torrijos, Ana Lucena-Varela, Eva Román, Elisabet Sánchez, Rubén Sánchez-Aldehuelo, Julia López-Cardona, Dhaarica Jeyanesan, Alejandro Esquivel Morocho, Itzel Canas-Perez, Christine Eastgate, Simone Di Cola, Lucia Lapenna, Giacomo Zaccherini, Deborah Bongiovanni, Antonio Riva, Rajni Sharma, Hio Lam Phoebe Tsou, Nicola Harris, Paola Zanaga, Katia Sayaf, Sabir Hossain, Javier Crespo, Mercedes Robles-Díaz, Antonio Madejón, Helena Degroote, Marko Korenjak, Xavier Verhelst, Javier García-Samaniego, Raúl J. Andrade, Paula Iruzubieta, Gavin Wright, Paolo Caraceni, Manuela Merli, Vishal C. Patel, Amir Gander, Agustín Albillos, Germán Soriano, Maria Francesca Donato, David Sacerdoti, Pierluigi Toniutto, Maria Buti, Christophe Duvoux, Paolo Antonio Grossi, Thomas Berg, Wojciech G. Polak, Massimo Puoti, Anna Bosch-Comas, Luca S. Belli, Patrizia Burra, Francesco Paolo Russo, Minneke Coenraad, José Luis Calleja, Giovanni Perricone, Shilpa Chokshi, Marina Berenguer, Joan Clària, Richard Moreau, Javier Fernández, Vicente Arroyo, Paolo Angeli, Cristina Sánchez-Garrido, Javier Ampuero, Salvatore Piano, Emanuele Nicastro, Nathalie Rock, Debbie Shawcross, Lindsey Edwards, Frauke Mutschler, Anette Melk, Gautam Mehta, Ulrich Baumann, Rajiv Jalan

**Affiliations:** 1 Department of Paediatric Kidney, Liver, and Metabolic Diseases, Hannover Medical School, Hannover, Germany.; 2 Department of General Pediatrics, Adolescent Medicine and Neonatology, Medical Center-University of Freiburg, Faculty of Medicine, University of Freiburg, Freiburg, Germany.; 3 Department of Gastroenterology and Hepatology, Hospital Clínico Universitario de Valencia, Valencia, Spain.; 4 Institute of Liver Studies, King’s College Hospital NHS Foundation Trust, London, United Kingdom.; 5 European Foundation for the Study of Chronic Liver Failure (EF CLIF), Barcelona, Spain.; 6 Pediatric Hepatology, Gastroenterology and Transplantation, Hospital Papa Giovanni XXIII, Bergamo, Department of Medicine and Surgery, University of Milano-Bicocca, Milan, Italy.; 7 Gastroenterology, Hepatology and Pediatric Nutrition Unit, Department of pediatrics, Gynecology and Obstetrics, Swiss Pediatric Liver Center, Geneva University Hospitals, University of Geneva, Geneva, Switzerland.; 8 Hepatology, HBP Surgery and Transplantation, Hepatology and Liver Transplant Unit, IIS La Fe University Hospital, Valencia, Spain.; 9 CIBEREHD, Universidad de Valencia, Valencia, Spain.; 10 Infectious Diseases Unit, ASST Grande Ospedale Metropolitano Niguarda, Milan, Italy.; 11 Department of Medical Biotechnology and Translational Medicine, Postgraduate School of Clinical Pharmacology and Toxicology, University of Milan, Milan, Italy.; 12 Hepatology and Gastroenterology Unit, ASST Grande Ospedale Metropolitano Niguarda, Milan, Italy.; 13 Department of Gastroenterology and Hepatology, Hospital Universitario Puerta de Hierro Majadahonda, IDIPHIM, Madrid, Spain.; 14 Central Unit of Clinical Research and Clinical Trials, Hospital Universitario La Paz, IdiPaz, Madrid, Spain.; 15 CIBEREHD, Madrid, Spain.; 16 Liver Unit, Hospital Universitario Valle de Hebron, Barcelona, Spain.; 17 Department of Gastroenterology and Hepatology, Leiden University Medical Center Transplant Center, Leiden, The Netherlands.; 18 Laboratory of Molecular Virology, Leiden University Medical Center, Leiden University Center of Infectious Diseases (LUCID), Leiden, The Netherlands.; 19 European Reference Network (ERN)RARE-LIVER.; 20 Unit of Internal Medicine and Hepatology (UIMH), Department of Medicine—DIMED, University of Padova, Padova, Italy.; 21 Hepatology and Liver Transplantation Unit, Azienda Sanitaria Universitaria Integrata, University of Udine, Udine, Italy.; 22 Medicine Department, University of Valencia, Valencia, Spain.; 23 Azienda Ospedaiera Universitaria Integrata Verona, Verona, Italy.; 24 Division of Gastroenterology and Hepatology, Foundation IRCCS Ca’ Granda Ospedale Maggiore Policlinico, Milan, Italy.; 25 Hospital Universitario Virgen del Rocio, Sevilla. Instituto de Biomedicina de Sevilla, Universidad de Sevilla, Sevilla, Spain.; 26 Hospital de la Santa Creu i Sant Pau, Barcelona, Spain.; 27 EUI-Sant Pau School of Nursing, Barcelona, Spain.; 28 Servicio de Gastroenterología, Hospital Universitario Ramón y Cajal, Instituto Ramón y Cajal de Investigación Sanitaria (IRYCIS), Centro de Investigación Biomédica en Red de Enfermedades Hepáticas y Digestivas (CIBEREHD), Instituto Salud Carlos III, Madrid, Spain.; 29 Royal Free London NHS Foundation Trust, London, United Kingdom.; 30 Department of Translational and Precision Medicine, University of Rome Sapienza, Rome, Italy.; 31 Department of Medical and Surgical Sciences, University of Bologna, Bologna, Italy.; 32 Unit of Semeiotics, Liver and Alcohol-related Diseases, IRCCS Azienda Ospedaliero-Universitaria di Bologna, Bologna, Italy.; 33 Roger Williams Institute of Liver Studies, King’s College London & Foundation for Liver Research, London, United Kingdom.; 34 Department of Surgery, Oncology and Gastroenterology, University of Padova, Padova, Italy.; 35 Gastroenterology and Multivisceral Transplant Units, Azienda Ospedale Università’ di Padova, Padova, Italy.; 36 Mid & South Essex NHS Foundation Trust, Basildon, United Kingdom.; 37 Gastroenterology and Hepatology Department, Marqués de Valdecilla University Hospital, Santander, Spain.; 38 Clinical and Traslational Digestive Research Group, Instituto de Investigación Sanitaria Valdecilla (IDIVAL), Santander, Spain.; 39 Servicio de Aparato Digestivo, Hospital Universitario Virgen de la Victoria, Instituto de Investigación Biomédica de Málaga y Plataforma en Nanomedicina–IBIMA Plataforma Bionand, Universidad de Málaga, Málaga, Spain.; 40 Liver Unit, Hospital Universitario La Paz, CIBERehd, IdiPAZ, Universidad Autónoma de Madrid, Madrid, Spain.; 41 Department of Gastroenterology and Hepatology, Ghent University Hospital, Ghent, Belgium.; 42 Department of Gastroenterology and Hepatology, Universitair Ziekenhuis Brussel (UZ Brussel), Jette, Belgium.; 43 European Liver Patients’ Association.; 44 Liver Research Center Ghent, Ghent University, Ghent, Belgium.; 45 Department of Hepatology-Liver Transplant Unit, Henri Mondor Hospital-APHP, Paris Est University, Paris, France.; 46 Department of Medicine and Surgery, University of Insubria, Infectious and Tropical Diseases Unit, ASST Sette Laghi, Varese, Italy.; 47 European Association for the Study of the Liver.; 48 Division of HPB and Transplant Surgery, Department of Surgery, Erasmus MC Transplant Institute, University Medical Center Rotterdam, Rotterdam, The Netherlands.; 49 Infectious Diseases Niguarda Great Metropolitan Hospital, University of Milano Bicocca, Milan, Italy.; 50 Faculty of Health, University of Plymouth, United Kingdom.; 51 Hospital Clínic, Institut d’Investigacions Biomèdiques August Pi-Sunyer (IDIBAPS), Centro de Investigación Biomédica en Red (CIBERehd) and Universitat de Barcelona, Barcelona, Spain.; 52 Centre de Recherche sur l’inflammation (CRI), INSERM and Université Paris Cité, Paris, France.; 53 Service d’hépatologie, APHP, Hôpital Beaujon, Clichy, France.; 54 Liver Unit, Hospital Clínic, Universitat de Barcelona, Institut d’Investigacions Biomèdiques August Pi-Sunyer (IDIBAPS) and Centro de Investigación Biomèdica en Red (CIBEREHD), Barcelona, Spain.; 55 Pediatric Hepatology, Gastroenterology and Transplantation, Ospedale Papa Giovanni XXIII, Bergamo, Italy.; 56 School of Immunology and Microbial Sciences, Faculty of Life Sciences and Medicine, Roger Williams Institute of Liver Studies, King’s College London, London, United Kingdom.; 57 Centre for Host Microbiome Interactions, Faculty of Dentistry, Oral and Craniofacial Sciences, King’s College London, London, United Kingdom.; 58 Institute for Liver and Digestive Heath, University College London, London, United Kingdom.; 59 Institute of Immunology and Immunotherapy, University of Birmingham, United Kingdom.

## Abstract

**Background.:**

Adult solid organ transplant recipients (SOTRs) have decreased responsiveness to severe acute respiratory syndrome coronavirus type 2 (SARS-CoV-2) vaccination and higher incidence of infection, but there are few data on the serological response in pediatric SOTR. The aim of this study was to determine serological response to SARS-CoV-2 vaccination in pediatric liver (LT) and kidney transplant (KT) recipients and compare it with adult SOTR.

**Methods.:**

A European, prospective, multicenter study was performed. Samples were taken at 7 and 32 wk following COVID-19 vaccination and serological endpoints were measured by ELISA.

**Results.:**

A total of 42 pediatric (16 post-LT and 26 post-KT) and 117 adult (all post-LT) were included. All pediatric participants and 94% adult participants received mRNA vaccines. Paediatric SOTR patients had significantly higher anti-Spike IgG levels than adult participants at week 7 (114 220.7 [59 285.92–220 058.55] versus 8756.7 [5643.69–13 586.71], *P* < 0.0001) and week 32 (46 113.2 [10 992.91–193 436.14] versus 8207.0 [3561.20–18 913.43], *P* = 0.0032). No significant difference in week 7 anti-Spike IgG response was found between pediatric LT and KT (129 434.4 [51 888.64–322 869.69] versus 105 304.5 [39 910.20–277 849.50], *P* = 0.9854). No differences were seen between children and adults in the rate of decline of anti-Spike IgG between weeks 7 and 32 (*P* = 0.8000). Male sex and hemolytic-uremic syndrome or postischemic kidney disease were associated with lower anti-Spike IgG levels at week 7 in pediatric SOTR.

**Conclusions.:**

Paediatric SOTR demonstrate greater SARS-CoV-2 vaccine responses than comparable adult SOTR patients. These data support efficacy and safety of SARS-CoV-2 vaccination in child SOTR and may alleviate vaccine hesitancy in this patient group.

The introduction of vaccines against COVID-19 has reduced the number of infections, hospitalizations and deaths. This applies not only to the general population but also, in particular, to risk groups such as those with liver disease and after transplantation.^1,2^ In general, COVID-19 infections in children are often mild.^3,4^ The multisystem inflammatory syndrome in children was soon described following a COVID-19 infection. A systemic complication with persistent fever, inflammation, and organ dysfunction is similar to Kawasaki disease.^5,6^ Moreover, there is a risk for long COVID-19 with a prevalence up to 25% after infection.^7^ Vaccination may lower the risk for developing long COVID-19 in adults.^8^ There are good data that COVID-19 vaccines are immunogenic and effective in healthy children and adolescents.^9-11^ In May 2021, BNT162b2 COVID-19 vaccine was authorized for the vaccination of 12- to 15-y olds in the European Union (EU).^12^ Since November 2021, the vaccine has also been authorized at a lower dose for children aged between 5 and 11 y throughout the EU.^13^ However, compared with adults, little is known about the safety and efficacy of COVID-19 vaccines in pediatric patients with underlying medical conditions or who are immunosuppressed. Moreover, children after solid organ transplantation have a high risk for vaccine-preventable infection, but they are often not age-appropriate vaccinated.^14-16^

Even before COVID-19, vaccine hesitancy was a leading cause of underimmunization, both among parents and primary care physicians.^17^ Nevertheless, COVID-19 vaccine acceptance in pediatric transplant population is even worse.^18^ Many different factors influence COVID-19 vaccine hesitancy; in particular vaccine-specific factors with regard to safety and effectiveness can only be answered by appropriate studies.^19,20^

The primary aim of this study was to determine serological response to severe acute respiratory syndrome coronavirus type 2 (SARS-CoV-2) vaccination in pediatric patients after liver (LT) or kidney transplant (KT) and undertake comparative analyses with adult patients after liver transplantation. Secondary aims were to evaluate rate of decline of serological response between weeks 7 and 32 in pediatric and adult patients and evaluate factors associated with serological response to SARS-CoV-2 vaccination in the pediatric group.

## MATERIALS AND METHODS

### Study Design and Participants

**FIGURE 1.**
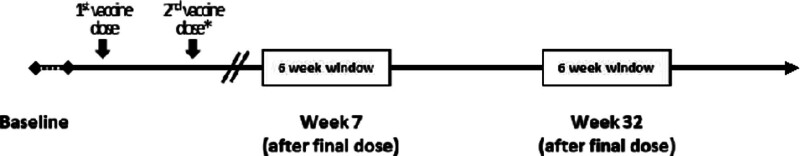
Study outline. *Second vaccine dose will be given after 6 wk (±1 wk after initial dose according to the recommendations for mRNA vaccines).

COBALT is a European, prospective, multicenter study of SARS-CoV-2 vaccine responses in adult and pediatric patients. Part of the adult data have been previously published.^2^ The study design is outlined in Figure 1, and the protocol is available (**Supplemental Material, SDC**). Recruitment for COBALT took place in Italy, Spain, and the United Kingdom. Inclusion criteria for the adult population were age 18 y or older and able to give written informed consent and post-LT (>6 mo) for cirrhosis. Exclusion criteria were history of COVID-19 (polymerase chain reaction–positive episode) or uncontrolled HIV infection. Inclusion criteria for the pediatric population were age between 0 and 17 y, whose parent/legal guardian give written informed consent and SOTR (≥1 y after pediatric LT or KT). Exclusion criteria were parents/legal guardian unable to give written informed consent, participant receiving immunoglobulin supplementation <3 mo, episode of biopsy-proven acute rejection <3 mo, participant receiving high dose steroids <3 mo, episode of posttransplant lymphoproliferative disease <3 mo, uncontrolled HIV infection, diagnosis of combined or severe combined immunodeficiency, or stem cell transplantation. Patients were assessed for eligibility for the study, in clinic or by telephone, at any point until 10 wk after COVID-19 vaccination from May 8, 2021, to September 10, 2021, in the case of adults and from June 14, 2021, to February 3, 2022, for pediatric population with clinical follow-up until June 1, 2024.

### Data Collection and Biological Sampling

The following information was collected at the time of inclusion: demographic data (date of birth, sex, race and ethnicity); medical history (date of onset/diagnosis and etiology of liver or kidney disease); medications and vaccine regimen received by participants (mRNA vaccines—2 doses of BNT162b2 Pfizer-BioNTech or mRNA-1273 Moderna, adenoviral vaccines—1 dose of Ad26.COV2.S Janssen/Johnson & Johnson or 2 doses of AZD1222 Oxford-AstraZeneca or heterologous combinations). Data were entered electronically into a predesigned electronic Case Report Form, maintained by the EF-CLIF Data Management Center.

All participants underwent blood sampling at 7 ± 3 and 32 ± 3 wk following second vaccine dose (or initial vaccine dose for 1-dose regimens) for laboratory analyses. Immunological assays were conducted at the King’s College London, United Kingdom. Hematology (full blood count and coagulation) and biochemistry (liver and renal function) profiles were processed at the local center and the results were clinician-reported.

### Laboratory Methods

#### Anti-Spike and Receptor-binding Domain IgG Immunoassays

IgG levels of SARS-CoV-2 spike and SARS-CoV-2 S1 receptor-binding domain (RBD) were determined in serum using electrochemiluminescent immunoassay from meso-scale discovery (V-PLEX COVID-19 CoV Panel 3 Kit, K15399U; Meso Scale Diagnostic, MD). According to the manufacturer’s instructions, serum samples were diluted 1:5000 before quantification. Assays were carried out without modification. Before analysis, data were normalized by log10 transformation.

### Statistical Analyses

#### Descriptive Analyses and Humoral Responses to COVID-19 Vaccination

Discrete variables were reported as counts (percentage), continuous variables normally distributed as mean and SD, and not-normally distributed as median and interquartile range (25th percentile–75th percentile). In univariable statistical comparisons, associations between categorical variables were tested using the Pearson chi-square test or Log-linear models depending on data complexity. Concentrations of anti-Spike/RBD IgM, IgG and IgG/IgM ratios at weeks 7 ± 3 and 32 ± 3 following COVID-19 vaccination were normalized by log10-transformation and presented as geometric mean (IU) and 95% confidence interval. The rate of decline was calculated as the difference of the log-transformed antibody levels between weeks 7 and 32. The Wilcoxon-Mann-Whitney or the Kruskal-Wallis tests were used for univariable comparisons between adult and pediatric patients or between LT and KT recipients.

#### Factors Associated With COVID-19 Vaccine Response

To study independent associations of antibody response in the pediatric population, univariable general linear models were performed including demographic, clinical, drug-related, and biochemical data. Only covariates showing a clinical and statistical significance in univariable models, or participating as a confounding factor for the variable of interest, were included in the final step-wise multivariable models.

#### Software and Data Quality Assurance

Statistical analyses were carried out using SAS v 9.4, R v 4.1.0, SPSS v26/27 and SIMCA v15/v17 depending on package availability and functionality, with the cutoff for statistical significance set at 0.05.

#### Research Reproducibility Approach

All analyses were reproducibly performed and were hosted in the EF-CLIF repository, which is publicly available on demand.

### Ethics

The study was conducted in accordance with the recommendations for physicians involved in research on human participants adopted by the 18th World Medical Assembly, Helsinki 1964 as revised and recognized by governing laws and EU Directives. The study was approved by ethical review boards at all study sites. Each participant’s (including children and their legal guardians) consent to participate in the study was obtained after a full age-appropriate explanation was given. The right of the participant to refuse to participate in the study without giving reasons was respected.

## RESULTS

### Participants

A total of 42 pediatric SOTR (16 post-LT and 26 post-KT) and 125 adult post-LT recipients were recruited. All pediatric participants received mRNA vaccines (BNT162b2 Pfizer-BioNTech) and 117 (93.6%) adults received mRNA vaccines. Only the 117 adult patients after mRNA vaccination were included in the further analyses. Participant characteristics are presented in Table 1. A total of 56% and 62% of pediatric patients were male with a mean age of 9 (3) and 11 (3) y in the LT and KT groups, respectively, whereas in adult patients, 78% were men with a mean age of 60 (12) y. The vast majority of patients were White. The most prevalent liver disease in LT pediatric patients was biliary atresia (38%) followed by malignancy (19%), whereas the most prevalent kidney disease in KT patients was congenital abnormalities (54%) followed by glomerular disease (31%). The most prevalent etiology of cirrhosis in adults was hepatitis B or C (48%) and alcohol (28%). All LT children, 96% of LT adults and 89% of KT pediatrics received immunosuppressors, being calcineurin inhibitors the most frequently used in all groups (88%, 86%, and 85% in LT and KT, respectively).

**TABLE 1.** Baseline characteristics of the study population

Variable	Pediatric SOTR (n = 42)	Pediatric post-LT (n = 16)	Pediatric post-KT (n = 26)	Adults post-LT (n = 117)
Male sex, n (%)	25 (60)	9 (56)	16 (62)	94 (80)
Age (y), mean (SD)	10 (3)	9 (3)	11 (3)	61 (12)***
Race, n (%) White Black or Afro-American	41 (98) 1 (2)	15 (94) 1 (6)	26 (100) 0 (0)	117 (100) 0 (0)
Ethnicity, n (%) North European Mediterranean Other	31 (76) 8 (20) 2 (5)	9 (60) 5 (33) 1 (7)	22 (85) 3 (12) 1 (4)	4 (3)*** 112 (96) 1 (1)
Liver disease, n (%) Biliary atresia Malignancy Cryptogenic Hemochromatosis Other		6 (38) 3 (19) 2 (13) 1 (6) 2 (13)		
Etiology of cirrhosis Alcohol NAFLD/NASH Autoimmune Hepatitis B or C Other				35 (30) 9 (8) 13 (11) 56 (48) 29 (28)
Years since the diagnosis of liver disease 1–5 >5		5 (33) 10 (67)		13 (13) 89 (87)
Kidney disease, n (%) Congenital abnormalities Glomerular disease Tubulo interstitial HUS/Postischemic Other			14 (54) 8 (31) 1 (4) 1 (4) 3 (12)	
Immunosuppressors, n (%) Steroids Calcineurin inhibitor Mycophenolate AZA or 6-MP Other	39 (93) 15 (36) 26 (86) 3 (7) 1 (2) 23 (55)	16 (100) 4 (25) 14 (88) 0 (0) 0 (0) 4 (25)	23 (89) 11 (42) 22 (85) 3 (12) 1 (4) 19 (73)^^	112 (96) 13 (11) 99 (85) 52 (44)*** 1 (1) 19 (16)
Biochemical parameters, mean (SD) or median (IQR) Albumin (g/dL) AST (U/L) ALT (U/L) LDH (U/L) ALP (U/L) GGT (U/L) Bilirubin (mg/dL) Creatinine (mg/dL) Ferritin (ug/dL) Sodium (mEq/L) Glucose (mg/dL) C-reactive protein (mg/L) Hemoglobin (g/dL) Leucocyte (×10^9^ cells/L) Lymphocytes (×10^9^ cells/L) Monocytes (×10^9^ cells/L) Neutrophils (×10^9^ cells/L) Platelets (×10^3^ cells/L) INR Quick, n (%)	4.2 (3.9–4.5) 36 (28–44) 27 (22–42) 247 (45) 276 (104) 18 (13–69) 0.4 (0.3–0.7) 0.7 (0.5–1.1) 8.6 (3.6–75) 139 (138–140) 90 (85–101) 0.6 (0.6–1.7) 13 (11–13) 5.5 (4.6–7.9) 2.4 (1.1) 0.4 (0.3–0.6) 2.7 (1.9–3.5) 277 (106) 1.1 (0.1) 88 (15)	3.9 (0.5–4.2) 37 (32–44) 28 (22–42) 235 (44) 331 (76) 22 (15–55) 0.4 (0.3–0.6) 0.4 (0.3–0.6) 6 (3–12) 139 (138–139) 88 (84–98) 0.6 (0.6–1.7) 13 (12–14) 5.1 (4.0–8.3) 2.4 (1.5) 0.4 (0.3–0.6) 2.2 (1.7–3.1) 207 (86) 1.1(0.1) 88 (15)	4.4 (4.2–4.6)^^ 30 (25–42) 25 (21–43) 261 (45) 201 (92)^^ 17 (13–116) 0.6 (0.5–0.7) 1.0 (0.7–1.4)^^ 75 (48–163) 139 (138–140) 92 (86–119) — 12 (11–13)^ 5.7 (4.9–7.8) 2.4 (0.9) 0.4 (0.4–0.5) 2.9 (2.0–3.8) 317 (96)^^ — —	4.2 (4.0–4.5)** 23 (18–29)*** 19.5 (14–32.5)* 219.1 (66.9)*** 106.5 (70.8)*** 30 (17–72) 0.7 (0.5–1.1)* 1.0 (0.8–1.3)*** 26 (26–26)*** 141 (139–142)*** 99 (89–114)* 4.3 (1.2–52.5)* 14.4 (12.4–15.5) 5.7 (4.5–7.0) 1.7 (1.1) 0.5 (0.4–0.6) 3.2 (2.5–4.3)* 150 (118– 215) 1.1 (0.2) 94.2 (16.3)

Significant differences between pediatric and adult LT recipients were expressed as *P < 0.05, **P < 0.01, ***P < 0.001. Significant differences between pediatric LT and KT recipients were expressed as ^*P* < 0.05, ^^*P* < 0.01, ^^^*P* < 0.001.

6-MP, mercaptopurine; ALP, alkaline phosphatase; ALT, alanine transaminase; AST, aspartate transaminase; AZA, azathioprine; GGT, gamma-glutamyltransferase, gamma-GT; INR, international normalized ratio; IQR, interquartile range; KT, kidney transplant; LDH, lactate dehydrogenase; LT, liver transplant; NAFLD/NASH ; SOTR, solid organ transplant recipient.

### Humoral Immune Responses to COVID-19 Vaccination

**FIGURE 2.**
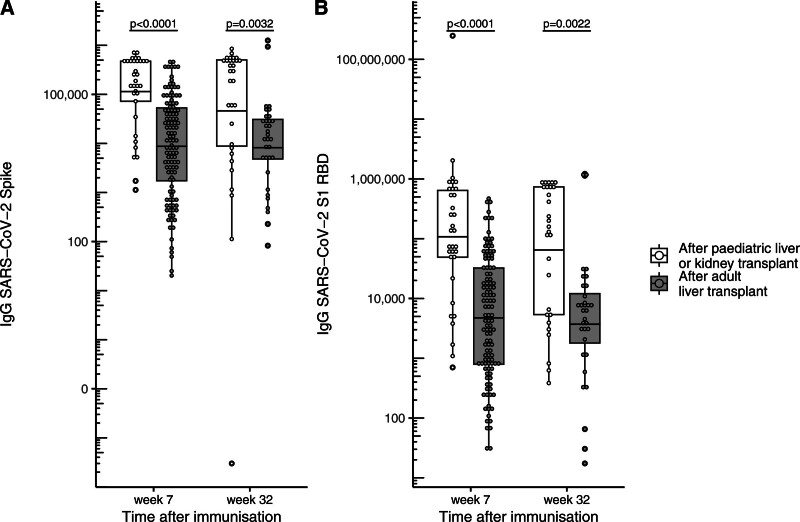
Humoral immune responses to COVID-19 vaccination in pediatric and adult patients. Comparison of anti-Spike IgG (A) and anti-RBD IgG (B) levels between pediatric and adult SOTR at weeks 7 and 32 following vaccination. Box plots show geometric mean and interquartile range. The y-axis is scaled logarithmically. RBD, receptor binding domain; SARS-CoV-2, severe acute respiratory syndrome coronavirus type 2; SOTR, solid organ transplant recipients.

Pediatric SOTR had significantly higher geometric mean anti-Spike IgG levels than adult participants at week 7 postvaccination (114 220.7 [59 285.92–220 058.55] versus 8756.7 [5643.69–13 586.71], *P* < 0.0001) and week 32 (46 113.2 [10 992.91–193 436.14] versus 8207.0 [3561.20–18 913.43], *P* = 0.0032) (Figure 2A). Similar findings were noted for anti-RBD IgG response at week 7 (107 718.8 [43 358.31–267 615.10] versus 4730.2 [3067.76–7293.63), *P* < 0.0001) and week 32 (64 760.8 [23 545.18–178 124.01] versus 3717.3 [1391.62–9929.75], *P* = 0.0022) (Figure 2B).

**FIGURE 3.**
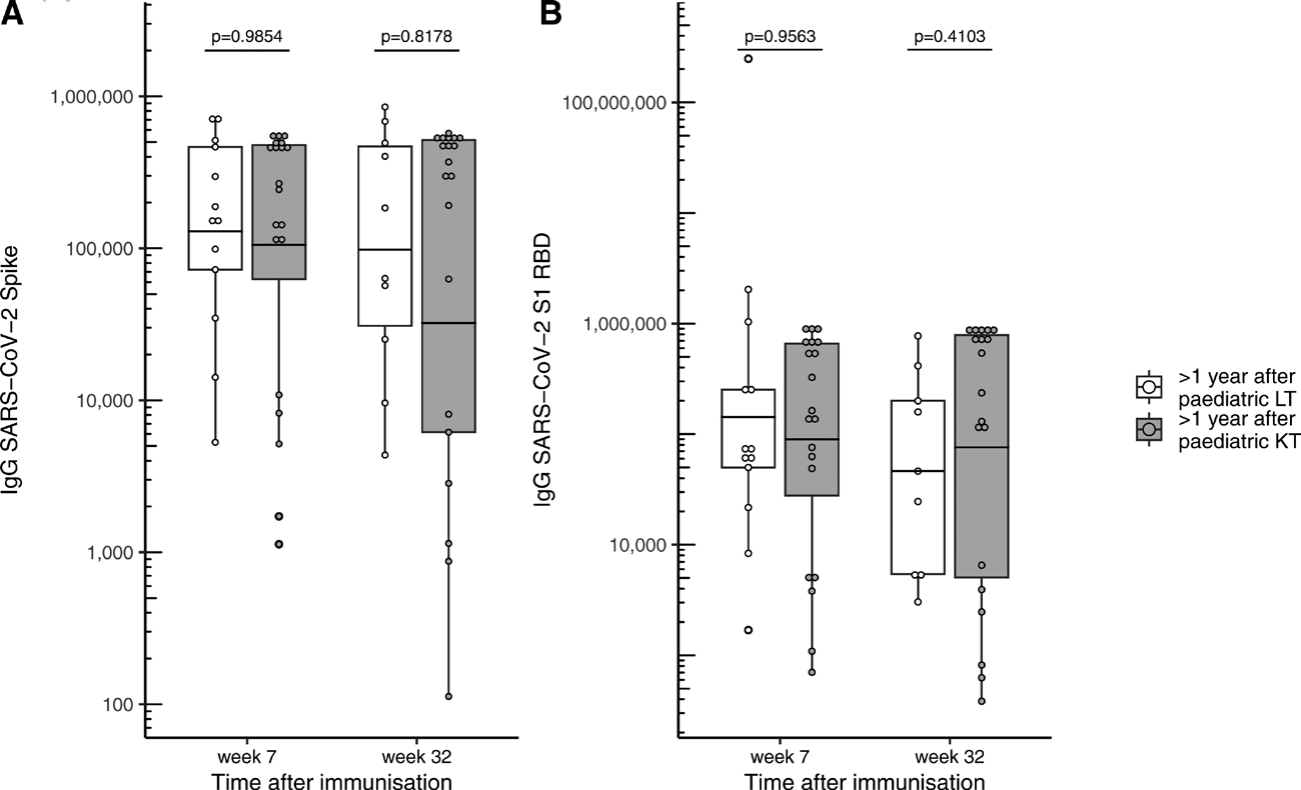
Humoral immune responses to COVID-19 vaccination in pediatric liver and kidney transplant patients. Comparison of anti-Spike IgG (A) and anti-RBD IgG (B) levels between pediatric LT and KT recipients. Box plots show geometric mean and interquartile range. The y-axis is scaled logarithmically. KT, kidney transplant; LT, liver transplant; RBD, receptor binding domain; SARS-CoV-2, severe acute respiratory syndrome coronavirus type 2.

Within the pediatric cohort, no significant difference in anti-Spike IgG response was found between LT and KT recipients at week 7 (129 434.4 [51 888.64–322 869.69] versus 105 304.5 [39 910.20–277 849.50], *P* = 0.9854) (Figure 3) or week 32 (97 912.0 [26 033.09–368 252.47] versus 32 218.5 [4009.23–258 910.92], *P* = 0.8178) (Figure 3A). Similar findings were noted for anti-RBD IgG response at week 7 (142 814.8 [24 521.05–831 778.51] versus 89 676.4 [29 528.01–272 346.82], *P* = 0.9563) and week 32 (46 422.5 [9617.05–224 086.16] versus 75 822.7 [18 964.97–303 142.27], *P* = 0.4103) (Figure 3B).

### Factors Associated With Antibody Levels in Pediatric Patients

Male sex and hemolytic-uremic syndrome or postischemic kidney disease were associated with lower anti-Spike IgG levels at week 7 in pediatric SOTR (Table 2).

**TABLE 2.** Factors associated with anti-Spike IgG at week 7 in pediatric SOTR

Variable	Estimate	95% CI	*P*
Male sex	−0.64	−1.17 to −0.11	**0.0200**
Age, y	0.04	−0.03 to 0.12	0.2518
Race, Black or Afro-American	−0.94	−2.59 to 0.72	0.2580
Ethnicity North European Mediterranean	0.07 0.32	−1.17to1.31 −1.06to1.69	0.9101 0.6390
Liver disease Biliary atresia Malignancy Cryptogenic Hemochromatosis Other	−0.13 0.82 −1.38 0.14 0.17	−0.88 to 0.61 −0.14 to 1.78 −2.99 to 0.24 −1.55 to 1.83 −1.04 to 1.39	0.7164 0.0918 0.0919 0.8655 0.7722
Years since the diagnosis of liver disease (1–5)	−0.24	−1.14 to 0.65	0.5584
Kidney disease, n (%) Congenital abnormalities Glomerular disease Tubulo interstitial HUS/postischemic Other	0.11 −0.08 0.70 −1.88 −0.56	−0.52 to 0.74 −0.79 to 0.63 −0.97 to 2.37 −3.42 to −0.33 −1.76 to 0.64	0.7180 0.8203 0.3999 **0.0187** 0.3483
Immunosuppressors, n (%) Steroids Calcineurin inhibitor Mycophenolate AZA or 6-MP Other	0.37 −0.07 −0.06 0.37 0.64 −0.05	−0.84 to 1.58 −0.68 to 0.55 −0.95 to 0.82 −0.83 to 1.58 −1.04 to 2.31 −0.63 to 0.53	0.5372 0.8230 0.8867 0.5311 0.4430 0.8584
Biochemical parameters, mean (SD) or median (IQR) Albumin (g/dL) AST (U/L) ALT (U/L) ALP (U/L) GGT (U/L) Bilirubin (mg/dL) Creatinine (mg/dL) Sodium (mEq/L) C-reactive protein (mg/L) Hemoglobin (g/dL) Leucocyte (×10^9^ cells/L) Lymphocytes (×10^9^ cells/L) Monocytes (×10^9^ cells/L) Neutrophils (×10^9^ cells/L) Platelets (×10^3^ cells/L) INR	−0.00 −0.01 −0.01 0.00 0.00 0.06 −0.00 −0.03 −0.12 −0.03 0.04 0.03 −0.07 0.09 −0.00 0.39	−0.23 to 0.23 −0.03 to 0.01 −0.02 to 0.00 −0.00 to 0.00 −0.00 to 0.01 −0.84 to 0.96 −0.17 to 0.16 −0.22 to 0.16 −0.51 to 0.28 −0.08 to 0.03 −0.08 to 0.15 −0.24 to 0.29 −1.24 to 1.10 −0.12 to 0.31 −0.00 to 0.00 −4.34 to 5.12	0.9703 0.3031 0.2015 0.9448 0.6403 0.8888 0.9698 0.7432 0.5041 0.3569 0.5358 0.8395 0.9055 0.3892 0.9826 0.8541

6-MP, mercaptopurine; ALP, alkaline phosphatase; ALT, alanine transaminase; AST, aspartate transaminase; AZA, azathioprine; GGT, gamma-glutamyltransferase, gamma-GT; INR, international normalized ratio; IQR, interquartile range; KT, kidney transplant; LDH, lactate dehydrogenase; LT, liver transplant; NAFLD/NASH ; SOTR, solid organ transplant recipient.

Bold *P* values denote statistical significance, with the threshold for significance set at 0.05.

Only malignancy as liver disease etiology for liver transplantation in children was associated with anti-RBD IgG levels at week 7 (estimate 1.84; 95% CI, 0.61-3.06; *P* = 0.0046).

### Rate of Decline of Serological Response

No differences were seen between children and adults (*P* = 0.5849), pediatric and adult LT recipients (*P* = 0.6844) or between LT and KT pediatric recipients (*P* = 0.8017), in the rate of decline of anti-Spike IgG between weeks 7 and 32. Similarly, no differences were seen between children and adults (*P* = 0.9369), or between LT and KT pediatric recipients (*P* = 0.0868), in the rate of decline of anti-Spike RBD between weeks 7 and 32.

Factors associated with anti-Spike IgG degradation in pediatric SOTR are shown in Table 3. C-reactive protein and treatment with immunosuppressors were associated with anti-Spike IgG decline between weeks 7 and 32 in children. An increase in C-reactive protein was associated with a reduction in the mean difference between weeks 7 and 32 (estimate −0.95; 95% CI, −1.58 to −0.33; *P* = 0.0133). Treatment with immunosuppressors (estimate, −4.24; 95% CI, −6.33 to −2.15; *P* = 0.0004) and more specifically treatment with calcineurin inhibitor (estimate, −3.05; 95% CI, −4.97 to −1.12; *P* = 0.0034) were associated with less anti-Spike IgG degradation between weeks 7 and 32 in pediatric patients.

**TABLE 3.** Factors associated with anti-Spike IgG decline between weeks 7 and 32 in pediatric SOTR

Variable	Estimate	95% CI	*P*
Male sex	−0.21	−1,81 to 1.39	0.7895
Age, y	0.11	−0.24 to 0.46	0.5102
Ethnicity North European Mediterranean	0.42 0.50	−3.69 to 4.53 −4.13 to 5.12	0.8343 0.8253
Liver disease Biliary atresia Malignancy Cryptogenic	−0.26 0.55 −2.59	−2.61 to 2.08 −1.78 to 2.88 −6.30 to 1.12	0.8181 0.6299 0.1617
Years since the diagnosis of liver disease (1–5)	−0.79	−3.36 to 1.78	0.4669
Immunosuppressors, n (%) Steroids Calcineurin inhibitor Mycophenolate AZA or 6-MP Other	−4.24 −0.92 −3.05 −0.42 −0.38 −0.87	−6.33 to −2.15 −2.47 to 0.63 −4.97 to −1.12 −4.30 to 3.46 −4.26 to 3.50 −2.39 to 0.66	**0.0004** 0.2309 **0.0034** 0.8258 0.8404 0.2521
Biochemical parameters, mean (SD) or median (IQR) Albumin (g/dL) AST (U/L) ALT (U/L) ALP (U/L) GGT (U/L) Bilirubin (mg/dL) Creatinine (mg/dL) Sodium (mEq/L) C-reactive protein (mg/L) Hemoglobin (g/dL) Leucocyte (×10^9^ cells/L) Lymphocytes (×10^9^ cells/L) Monocytes (×10^9^ cells/L) Neutrophils (×10^9^ cells/L) Platelets (×10^3^ cells/L) INR	−0.18 −0.02 −0.01 −0.00 −0.00 0.87 0.18 0.28 −0.95 0.13 0.02 0.08 0.20 −0.02 −0.00 −9.57	−0.65 to 0.29 −0.06 to 0.01 −0.03 to 0.01 −0.01 to 0.01 −0.01 to 0.01 4.09 to 5.84 −0.08 to 0.43 −0.04 to 0.59 −1.58 to −0.33 −0.30 to 0.55 −0.21 to 0.25 −0.46 to 0.62 −1.92 to 2.33 −0.41 to 0.36 −0.01 to 0.00 −36.3 to 17.12	0.4236 0.1429 0.4266 0.7526 0.6715 0.6813 0.1715 0.0823 **0.0133** 0.5457 0.8415 0.7578 0.8445 0.8953 0.6130 0.3367

6-MP, mercaptopurine; ALP, alkaline phosphatase; ALT, alanine transaminase; AST, aspartate transaminase; AZA, azathioprine; GGT, gamma-glutamyltransferase, gamma-GT; INR, international normalized ratio; IQR, interquartile range; KT, kidney transplant; LDH, lactate dehydrogenase; LT, liver transplant; NAFLD/NASH ; SOTR, solid organ transplant recipient.

Bold *P* values denote statistical significance, with the threshold for significance set at 0.05.

## DISCUSSION

This study, as part of the COBALT initiative, adds to the growing body of evidence regarding COVID-19 vaccination in adult and pediatric SOTRs. These data remain of great importance, even in the postpandemic era, because of the issue of low vaccine coverage and vaccine hesitancy among this patient group and parents/guardians.^18^

The principal finding of this study is robust humoral immune response to initial SARS-CoV-2 vaccination in pediatric SOTRs. Specifically, we observed a significantly higher anti-Spike and anti-RBD IgG response in pediatric SOTRs compared with adult SOTRs at both 7- and 32-wk postvaccination. Both pediatric and adult patients had similar rates of anti-Spike IgG degradation during the follow-up period of 8 mo (32 wk) following initial vaccination. In terms of degree of humoral response to vaccination, male sex and history of hemolytic-uremic syndrome or postischemic kidney disease were associated with decreased anti-Spike IgG levels at week 7. It is notable too that age and maturation of the immune system appears to be the dominant determinant to vaccine response and not the different level of immunosuppression that is used between pediatric liver and kidney recipients.

Very few studies have measured SARS-CoV-2 vaccine responses in pediatric liver transplant recipients, and to our knowledge, this is the first study to compare responses between pediatric and adult liver transplant recipients with similar characteristics. Several studies have evaluated SARS-CoV-2 vaccine responses in pediatric renal transplant recipients; a systematic review of these data demonstrated that rituximab administration, mycophenolate mofetil therapy and lower GFR reduce the vaccination response rates.^21-28^ By contrast, there is only 1 published series reporting SARS-CoV-2 vaccine responses in pediatric liver transplant recipients, with no adult comparator group or longitudinal follow-up or response.^29^

Although recommendations for precision medicine approaches cannot be made from the small sample sizes reported here, these data are consistent with greater SARS-CoV-2 vaccine responses seen in healthy children and adolescents compared with adults to mRNA vaccines.^9^ Similar findings were also noted in a Chinese study comparing responses to Ad5-vectored SARS-CoV-2 vaccine response in pediatric and adult recipients.^30^

There are several limitations to the current study. First, these data were collected in a real-world observational cohort, and therefore, the analyses are largely descriptive without any prespecified sample size calculation or analysis plan. This also includes that possible COVID-19 infections occurring before or after vaccination may influence the immune response in this observational cohort. As noted earlier, the sample sizes are small, and consequently, recommendations for precision medicine approaches or policy change cannot be made. There is little ethnic or racial diversity among the pediatric cohort, although in older age groups vaccine efficacy has been shown to be similar between races and ethnicities. Finally, there is no efficacy data presented here. Nevertheless, these data are scarce, and represent the only cohort reporting post-LT SARS-CoV-2 vaccine responses in pediatric patients outside China, and the only cohort with longitudinal data collection. Moreover, this is the only study in this patient group to report vaccine responses with a comparable adult cohort recruited alongside.

There remain several areas to research within the field of SARS-CoV-2 vaccination within this pediatric patient group. Specifically, it remains unclear: what is the optimal vaccine schedule for pediatric SOTRs; should booster doses be routinely administered, and if so, when; which immunosuppressive regimens are associated with the best vaccine response; what is the role of cellular immunity in protection against COVID-19 in this population? Nevertheless, these data may shed light on the difference in immune response between children and adults and aid in future development of vaccines for immunocompromised adults and children. This is important to combat vaccine hesitancy and low coverage among SOTR and families for SARS-CoV-2 and potentially future viral pandemics.

## References

[1] Ballester MP Jalan R Mehta G. Vaccination in liver diseases and liver transplantation: Recommendations, implications and opportunities in the post-covid era. JHEP Rep. 2023;5:100776. 37360567 10.1016/j.jhepr.2023.100776 PMC10241163

[2] Mehta G Riva A Ballester MP; COBALT Consortium. Serological response and breakthrough infection after COVID-19 vaccination in patients with cirrhosis and post-liver transplant. Hepatol Commun. 2023;7:e0273. 37870985 10.1097/HC9.0000000000000273 PMC10586829

[3] Flaxman S Whittaker C Semenova E. Assessment of COVID-19 as the underlying cause of death among children and young people aged 0 to 19 years in the US. JAMA Netw Open. 2023;6:e2253590. 36716029 10.1001/jamanetworkopen.2022.53590 PMC9887489

[4] Zimmermann P Curtis N. Why does the severity of COVID-19 differ with age?: understanding the mechanisms underlying the age gradient in outcome following SARS-CoV-2 infection. Pediatr Infect Dis J. 2022;41:e36–e45. 34966142 10.1097/INF.0000000000003413 PMC8740029

[5] Riphagen S Gomez X Gonzalez-Martinez C. Hyperinflammatory shock in children during COVID-19 pandemic. Lancet. 2020;395:1607–1608. 32386565 10.1016/S0140-6736(20)31094-1 PMC7204765

[6] Verdoni L Mazza A Gervasoni A. An outbreak of severe Kawasaki-like disease at the Italian epicentre of the SARS-CoV-2 epidemic: an observational cohort study. Lancet. 2020;395:1771–1778. 32410760 10.1016/S0140-6736(20)31103-X PMC7220177

[7] Lopez-Leon S Wegman-Ostrosky T Ayuzo Del Valle NC. Long-COVID in children and adolescents: a systematic review and meta-analyses. Sci Rep. 2022;12:9950. 35739136 10.1038/s41598-022-13495-5 PMC9226045

[8] Watanabe A Iwagami M Yasuhara J. Protective effect of COVID-19 vaccination against long COVID syndrome: a systematic review and meta-analysis. Vaccine. 2023;41:1783–1790. 36774332 10.1016/j.vaccine.2023.02.008 PMC9905096

[9] Frenck RW Jr Klein NP Kitchin N; C4591001 Clinical Trial Group. Safety, immunogenicity, and efficacy of the BNT162b2 Covid-19 vaccine in adolescents. N Engl J Med. 2021;385:239–250. 34043894 10.1056/NEJMoa2107456 PMC8174030

[10] Munoz FM Sher LD Sabharwal C; C4591007 Clinical Trial Group. Evaluation of BNT162b2 Covid-19 vaccine in children younger than 5 years of age. N Engl J Med. 2023;388:621–634. 36791162 10.1056/NEJMoa2211031 PMC9947923

[11] Walter EB Talaat KR Sabharwal C; C4591007 Clinical Trial Group. Evaluation of the BNT162b2 Covid-19 vaccine in children 5 to 11 years of age. N Engl J Med. 2022;386:35–46. 34752019 10.1056/NEJMoa2116298 PMC8609605

[12] European Medicines Agency. First COVID-19 vaccine approved for children aged 12 to 15 in EU. Available at https://www.ema.europa.eu/en/news/first-covid-19-vaccine-approved-children-aged-12-15-eu. Accessed July 18, 2024.

[13] European Medicines Agency. Comirnaty COVID-19 vaccine: EMA recommends approval for children aged 5 to 11. Available at https://www.ema.europa.eu/en/news/comirnaty-covid-19-vaccine-ema-recommends-approval-children-aged-5-11. Accessed July 18, 2024.

[14] Feldman AG Beaty BL Curtis D. Incidence of hospitalization for vaccine-preventable infections in children following solid organ transplant and associated morbidity, mortality, and costs. JAMA Pediatr. 2019;173:260–268. 30640369 10.1001/jamapediatrics.2018.4954 PMC6439884

[15] Feldman AG Sundaram SS Beaty BL. Immunization status at the time of liver transplant in children and adolescents. JAMA. 2019;322:1822–1824. 31714979 10.1001/jama.2019.14386 PMC6865293

[16] Laue T Demir Z Debray D. Under-vaccination in pediatric liver transplant candidates with acute and chronic liver disease—a retrospective observational study of the european reference network transplantchild. Children (Basel). 2021;8:675. 34438566 10.3390/children8080675 PMC8394134

[17] Feldman AG Kempe A Beaty BL; Studies of Pediatric Liver Transplantation (SPLIT) Research Group. Immunization practices among pediatric transplant hepatologists. Pediatr Transplant. 2016;20:1038–1044. 27449120 10.1111/petr.12765

[18] Zheng Z Lu Y Wang M. Low COVID-19 vaccine coverage and guardian acceptance among pediatric transplant recipients. J Med Virol. 2023;95:e28377. 36478241 10.1002/jmv.28377 PMC9877554

[19] Adu P Poopola T Medvedev ON. Implications for COVID-19 vaccine uptake: a systematic review. J Infect Public Health. 2023;16:441–466. 36738689 10.1016/j.jiph.2023.01.020 PMC9884645

[20] Kafadar AH Tekeli GG Jones KA. Determinants for COVID-19 vaccine hesitancy in the general population: a systematic review of reviews. Z Gesundh Wiss. 2022:1–17. 10.1007/s10389-022-01753-9 PMC9483252 36160668

[21] Crane C Phebus E Ingulli E. Antibody response to 2- and 3-dose SARS-CoV-2 mRNA vaccination in pediatric and adolescent kidney transplant recipients. Pediatr Nephrol. 2023;38:611–614. 35759003 10.1007/s00467-022-05661-8 PMC9244318

[22] Emmanouilidou-Fotoulaki E Karava V Dotis J. Immunologic response to SARS-CoV-2 vaccination in pediatric kidney transplant recipients: a systematic review and meta-analysis. Vaccines (Basel). 2023;11:1080. 37376469 10.3390/vaccines11061080 PMC10302704

[23] Cirillo L Citera F Mazzierli T. Response to third dose of vaccine against SARS-CoV-2 in adolescent and young adult kidney transplant recipients. Transplantation. 2022;106:e386–e387. 35581690 10.1097/TP.0000000000004199

[24] Kermond RF Ozimek-Kulik JE Kim S. Immunologic response to SARS-CoV-2 mRNA vaccination in pediatric kidney transplant recipients. Pediatr Nephrol. 2023;38:859–866. 35833990 10.1007/s00467-022-05679-y PMC9281214

[25] Haskin O Ashkenazi-Hoffnung L Ziv N. Serological response to the BNT162b2 COVID-19 mRNA vaccine in adolescent and young adult kidney transplant recipients. Transplantation. 2021;105:e226–e233. 34381004 10.1097/TP.0000000000003922 PMC8549126

[26] Sattler A Thumfart J Toth L. SARS-CoV2 mRNA vaccine-specific B-, T- and humoral responses in adolescents after kidney transplantation. Transpl Int. 2022;35:10677. 35992746 10.3389/ti.2022.10677 PMC9385879

[27] Gulmez R Ozbey D Agbas A. Humoral and cellular immune response to SARS-CoV-2 mRNA BNT162b2 vaccine in pediatric kidney transplant recipients compared with dialysis patients and healthy children. Pediatr Nephrol. 2023;38:2199–2208. 36459243 10.1007/s00467-022-05813-w PMC9716124

[28] Stich M Di Cristanziano V Tonshoff B. Humoral immune response and live-virus neutralization of the SARS-CoV-2 omicron (BA.1) variant after COVID-19 mRNA vaccination in children and young adults with chronic kidney disease. Pediatr Nephrol. 2023;38:1935–1948. 36409368 10.1007/s00467-022-05806-9 PMC9684918

[29] Zheng Z Wu H Sun X. Evaluation of the effectiveness and safety of sequential vaccination with inactivated SARS-CoV-2 vaccine and Ad5-nCoV booster in pediatric liver transplant recipients. J Med Virol. 2024;96:e29543. 38528839 10.1002/jmv.29543

[30] Zhu F Jin P Zhu T. Safety and immunogenicity of a recombinant adenovirus type-5-vectored Coronavirus Disease 2019 (COVID-19) vaccine with a homologous prime-boost regimen in healthy participants aged >/=6 years: a randomized, double-blind, placebo-controlled, phase 2b trial. Clin Infect Dis. 2022;75:e783–e791. 34551104 10.1093/cid/ciab845 PMC8522421

